# The Emergency Medical System in Greece: Opening Aeolus’ Bag of Winds

**DOI:** 10.3390/ijerph15040745

**Published:** 2018-04-13

**Authors:** Ourania S. Kotsiou, David S. Srivastava, Panagiotis Kotsios, Aristomenis K. Exadaktylos, Konstantinos I. Gourgoulianis

**Affiliations:** 1 Respiratory Medicine Department, Faculty of Medicine, University of Thessaly, Biopolis, 41500 Larissa, Greece; kgourg@med.uth.gr; 2 Inselspital, University Hospital Bern, 3010 Bern, Switzerland; DavidShiva.Srivastava@insel.ch (D.S.S.); Aristomenis.Exadaktylos@insel.ch (A.K.E.); 3 International Business Department, Perrotis College, 57001 Thessaloniki, Greece; panagiotiskotsios@gmail.com

**Keywords:** ambulance, economic recession, emergency medical service, Greece, primary healthcare system, refugee, triage

## Abstract

An Emergency Medical Service (EMS) system must encompass a spectrum of care, with dedicated pre-hospital and in-hospital medical facilities. It has to be organised in such a way as to include all necessary services—such as triage accurate initial assessment, prompt resuscitation, efficient management of emergency cases, and transport to definitive care. The global economic downturn has had a direct effect on the health sector and poses additional threats to the healthcare system. Greece is one of the hardest-hit countries. This manuscript aims to present the structure of the Greek EMS system and the impact of the current economic recession on it. Nowadays, primary care suffers major shortages in crucial equipment, unmet health needs, and ineffective central coordination. Patients are also facing economic limitations that lead to difficulties in using healthcare services. The multi-factorial problem of in-hospital EMS overcrowding is also evident and has been linked with potentially poorer clinical outcomes. Furthermore, the ongoing refugee crisis challenges the national EMS. Adoption of a triage scale, expansion of the primary care network, and an effective primary–hospital continuum of care are urgently needed in Greece to provide comprehensive, culturally competent, and high-quality health care.

## 1. Introduction

Emergency Medical Services (EMS) have been described as “a comprehensive system which provides the arrangements of personnel, facilities, and equipment for the effective, coordinated, and timely delivery of health and adequate and specialized services to victims of sudden illness or injury” [1]. High-quality EMS is a major factor in any healthcare system.

An EMS system encompasses a spectrum of care characterised by dedicated medical facilities at the pre-hospital and in-hospital levels. It has to be organised in such a way as to include all necessary services—such as triage accurate initial assessment, prompt resuscitation, efficient and effective medical management, and transport of emergency cases to definitive care [1].

However, recent estimates have shown that 24 million patients die each year in low- and middle-income countries due to unprepared pre-hospital and in-hospital medical systems [2]. In detail, it has been previously reported that, in high-income settings, 59% of trauma deaths that occurred at the pre-hospital level were deemed as preventable [3]. The rates increased further in the middle- and low-income prehospital environments (72% and 81%, respectively) [3,4]. On the other hand, globally, the prevalence and mortality of emergency conditions remain high [2,3,4,5]. The burden of disease that requires emergency management is higher in low-income countries [5]. Analytically, the median disability-adjusted life years (DALYs) burden has been estimated at 48,000, 25,000, and 16,000 per 100,000 people for low-, middle- and high-income countries respectively [5].

It is widely accepted that an economic recession may have a severe impact on the psychological well-being of the patient population and may act as a precipitator for suicide risk [6]. Furthermore, it can increase the burden of chronic diseases, for instance in case of chronic obstructive pulmonary disease [6]. An economic crisis may also impair the coronary health of vulnerable individuals [6]. Notably, it has been argued that an economic recession could pose negative consequences for both quality of care and patients’ outcomes [6]. It should be emphasised that emergency departments (EDs) are where society’s problems first show up, from drug abuse and domestic violence to the health problems of the poor and uninsured [7]. When the 2007 global economic downturn began, it spread to Europe rapidly and Greece was one of the hardest-hit countries. This manuscript aims to present the structure of the Greek pre-hospital and in-hospital emergency care and summarize the consequences of the current economic recession on the Greek EMS system. A computer-based search of the English literature was performed in PubMed and Scopus. The considerable body of information examined in this article includes data that are to a large extent, but not exclusively, presented in Greek studies.

## 2. Greek EMS Structure

### 2.1. Greek Pre-Hospital EMS Structure: Past and Present

Since the 1970s, prehospital emergency health care can mainly be divided into two distinct models. These are the “load and go” Anglo-American model versus the “delay and treat” Franco-German model [1]. Another EMS delivery system has attracted attention in the United Kingdom. According to this, the role of primary health care is strengthened, thus, increasing the percentage of patients who are treated in a pre-hospital community setting or at the scene of an incident [1]. These categorical distinctions were accurate until the end of the 20th century [1]. Today, most EMS systems around the world partially share features of all aforementioned models [1].

The Greek health system is cure-oriented. Αnglo-American EMS principles are mainly applied in Greece. Before 1985, the Greek Red Cross and the Samaritans were responsible for rendering healthcare services, otherwise medical care was provided privately. The creation of the national healthcare system (ESY, NHS) in 1983 constituted a milestone in establishing a Greek healthcare system [8].

In 1915, Greek EMS began with the creation of the first aid station, funded by “Sotir” (Saviour), a non-profit organization [8]. From 1932 to 1988, the Samaritans founded several emergency stations in Athens, Thessaloniki, and Patras and equipped them with some ambulances [8]. In 1965, primary health care expanded into four major stations in Athens, Piraeus, Thessaloniki, and Patras, founded by the Social Insurance Institute (IKA). These stations were equipped with four additional ambulances [8]. In 1987, the two previous providers of EMS in Greece, the Hellenic Red Cross and IKA, merged to become the Hellenic EMS (EKAB) [8]. This process also established “166” as the national emergency number. Since then, Greek EMS has been exclusively handled by EKAB, which is entirely funded by the government [8].

### 2.2. Structure of Pre-Hospital EMS in Greece

Pre-hospital EMS is composed of different services ranging from healthcare positions attended by active medical personnel to call centres (dispatch centres) attended by professionals that can determine and handle urgency, to offer medical advice and to urgently dispatch a mobile medical unit [8].

#### 2.2.1. EMS Dispatch System in Greece

**Figure 1:**
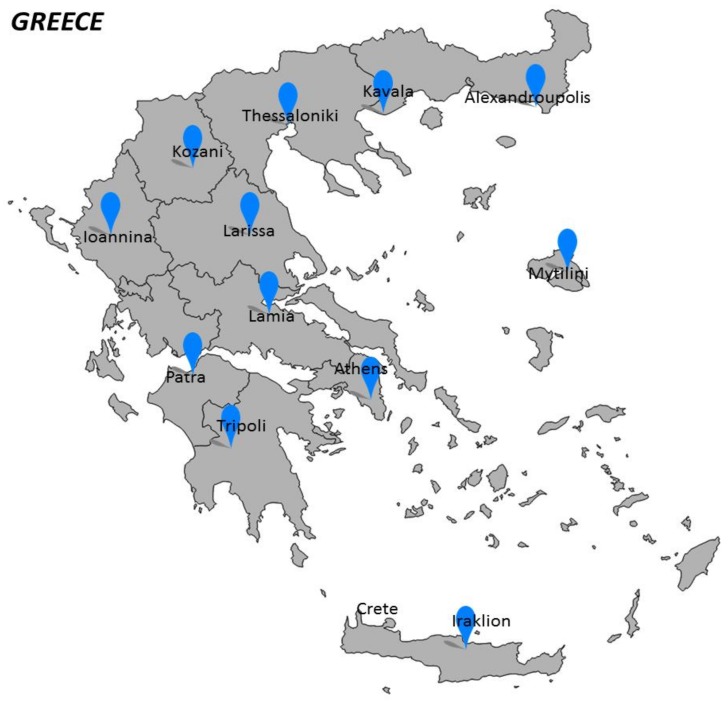
National Centres for Emergency Care (EKAB stations) in Greece. There are 12 EKAB stations in the major Greek cities: Athens, Thessaloniki, Patra, Iraklion (Crete), Larissa, Kavala, Ioannina, Alexandroupolis, Lamia, Mytilini, Tripoli, Kozani. Adapted from: https://www.ekab.gr/chorotaxiki-katanomi/ [9].

Today, EMS is managed via 12 EKAB stations in the major Greek cities (Figure 1) [8]. Several substations in smaller cities are controlled by a central station, so that a larger area can be supervised. Together this network covers 96.2% of the urban areas [8]. A local hospital coordinates and cooperates with local healthcare stations. It plays a crucial role in supporting primary care and acts as a gateway to more specialized care [8]. The EMS system supervises and provides advice to the EKAB stations [8].

**Figure 2:**
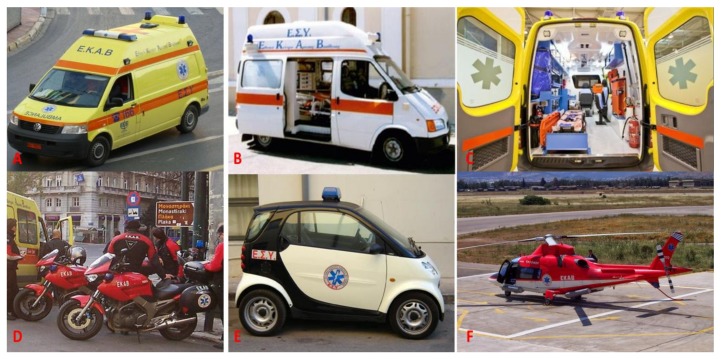
Types of EKAB ambulances. (**A**,**B**) Basic type of Ambulance; (**C**) Mobile Intensive Care Units; (**D**) Motorcycle ambulances; (**E**) Small vehicles for minor roads; (**F**) Helicopters.

Accordingly, EKAB stations and substations have their medical staff administrators and call centre [8]. Healthcare functions are traditionally carried out by physicians and emergency medical technicians (EMTs) [8]. Further, each station has its ambulances and medical equipment [8]. The EKAB possesses 740 basic life support ambulances, of which 502 are used by EKAB stations and 238 by hospitals and primary healthcare stations. Moreover, it holds 174 Mobile Intensive Care Units (MICUs), along with 25 motorcycles, 4 small vehicles for minor roads (smart, fast, saxos), three helicopters, 2 traffic coordination centres, and 2 vehicles modified for disaster recovery *(*Figure 2) [8].

Two types of ambulances are staffed by emergency personnel and are used for emergency transportations [8]. One type is the basic ambulance (Figure 2A,B) which has standard medical equipment for primary airway management procedures and oxygen delivery such as bag-mask ventilation, oxygen supplements, and a portable suction. Furthermore, it is equipped with an automated external defibrillator (AED), first aid wound care supplies, intravenous access kits, and immobilisation equipment. All ambulances are crewed by EMTs. This type of ambulance is the most commonly used [8].

The second type consists of MICUs (Figure 2C). These ambulances are equipped with more advanced equipment for skillful cardiopulmonary resuscitation, airway management, and adequate oxygenation, such as ventilators, pulse oximeters, a non-invasive transcutaneous pacemaker, a cardiac monitor, and defibrillators with (3-lead)-ECG-monitoring. MICUs are also fitted with intravenous access equipment, various devices for immobilization, as well as fluid replacement therapies in cases of hypovolemic shock and several drugs [8]. These units are mainly used in large cities like Athens and Thessaloniki and are staffed by a sole paramedic and an anesthesiologist [8]. Due to the heavy traffic conditions, these units can provide advanced life support much faster than an ambulance [8]. They initiate prehospital emergency care on the spot while waiting for the transport ambulance to arrive, thus saving valuable time when treating trauma patients [8].

The MICUs in Thessaloniki are also equipped with non-invasive positive pressure ventilation (Boussignac CPAP). They are certified to provide chest tube insertion and drainage as well as qualified management of difficult airways demanding intubation. Similarly, they are staffed both by a physician and EMTs [8].

Furthermore, in the five major cities (Athens, Thessaloniki, Patras, Larissa, and Iraklion), motorcycle ambulances are either single- or double-crewed. Specifically, they carry a solo paramedic (in Thessaloniki), or there is one physician with paramedic coverage (in Athens, Patras, Iraklion and Ioannina), respectively, to achieve a rapid response in the provision of medical care (Figure 2D) [8].

EKAB is also a significant aeromedical transportation provider, being the owner of three helicopters (Augusta A-109 Power) (Figure 2E) [8]. It has been recently estimated that approximately 500,000 patients are transported by EKAB per year. Furthermore, 2000 to 2500 medical flights were conducted per year [8].

**Figure 3:**
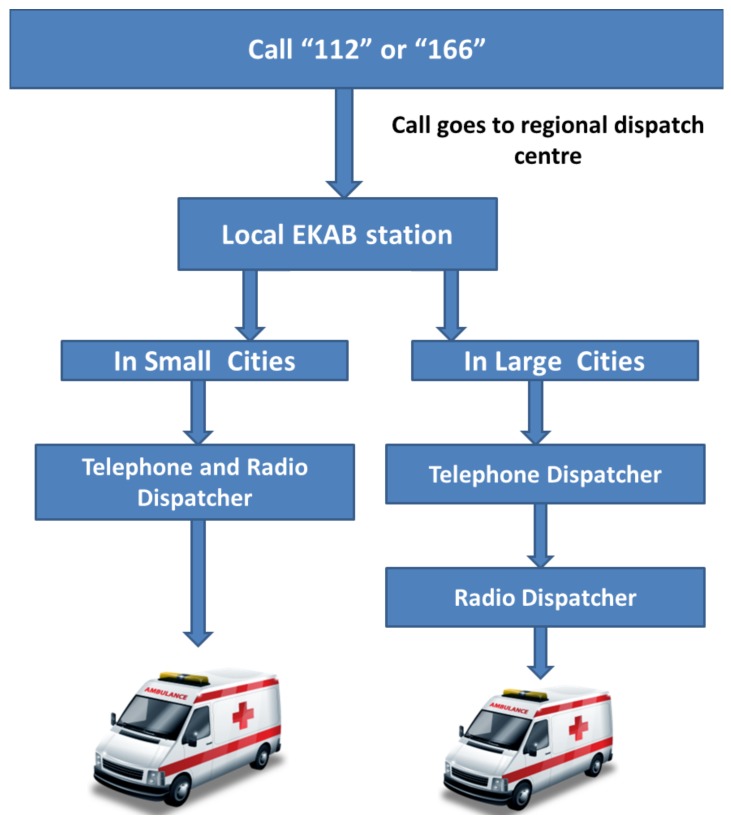
Dispatch process flowchart for Greece. All EKAB stations have their dispatch centres. EKAB is accessible throughout the country firstly by the European emergency phone number “112” as well as by the toll-free, easy to remember “166”. The call goes to the regional dispatch centre. A telephone dispatcher receives various calls and prioritises them according to importance or urgency. In large cities, telephone dispatchers mobilize the appropriate number of nearest available mobile ambulances or Mobile Intensive Care Units (MICUs) via radio communications and provide directions to arrive at the scene of the incident. On the contrary, in small cities, the dispatcher telephones the nearest ambulance station or else passes mobilization instructions to the radio operator if an ambulance is already mobile. Adapted from: Page C et al. [10].

Emergency medical support (EKAB) is accessible throughout the country firstly by the universal emergency phone number “112” as well as by the toll-free “166” (Figure 3) [8].

The dispatch centres consist of experienced operators (dispatchers) who triage phone calls; a complete telephone and radio recording system; and a computer network for data throughput, storage, and processing [10]. A properly implemented EMS telemedicine system is used for the guidance of aeromedical transportation [8]. EKAB dispatcher control stations handle emergency calls via telephone or radio communications [8,10]. The dispatchers receive calls from individuals, pre-hospital or in-hospital healthcare facilities, and other emergency agencies such as police or fire brigades [8]. They prioritize the urgency and decide where the case should be transferred. Besides, there is direct communication between the transferring and receiving facilities [8]. All dispatch centres are composed of physicians [8]. In case of a catastrophe, one of the major priorities of the dispatch centre is to coordinate healthcare institutes with other rescue services, such as the military, the police, and the firefighters [8]. Moreover, although the improvement of telemedicine systems has allowed wireless data transmission from moving vehicles, these technologies still are not widely used in Greek EMS system [1].

Remarkably, emergency medicine is not yet a recognised specialty in Greece. However, a Greek Society for Emergency Medicine was established in 2007 [8].

Furthermore, education and training centres were established in almost all EKAB stations in 1987 [8]. By 1989, all EMTs had received a 40 h training course [8]. In 1989, the Ministry of Health introduced a basic EMT training program [8]. Since 1995, four EKAB education centres have been founded in the biggest Greek cities (Athens, Thessaloniki, Patras, and Iraklion) and offered physicians a one-year course, comprising 400 h of training, 75 h of classroom-based training, 25 h of workshops, and 300 h of skills training. The training program is performed at EDs, Departments of Anesthesiology, adult and Neonatal Intensive Care Units, Cardiac Intensive Care Units and in the MICUs [8]. Currently, courses are organised into subjects that cover assessment of the critical illness and triage, airway skills, basic and advanced life support (BLS, ALS), resuscitation and advanced trauma life support (immobilization of injured bones and wound management) [8].

In 2000, a more advanced educational program was developed, establishing and developing a professional body recognised by national law [8]. This free of charge, two-year program offers extensive theoretical knowledge and practical experience through 1400 h of technical training, divided into 800 h of theoretical training and 600 h of clinical practice in hospitals and ambulances [8]. Furthermore, these programs offer courses in anatomy, physiology, pharmacology, pathology, ECG-monitoring, as well as training in methods of venous access, disaster management, established guidelines, safe driving, the English language, communication systems, and technologies and computer training [8]. Lastly, two semester-length training courses have been recently approved by the National Organization for the Certification of Qualifications and Vocational Guidance (EOPPEP), a national authority responsible for lifelong learning services [8]. After having completed each of the aforementioned courses, recipients obtain the diploma of “Adequacy in Prehospital Emergency Medicine” [8]. More anesthetists and fewer cardiologists were among the first physicians who were employed by EKAB services and National Health System [8].

#### 2.2.2. Primary Healthcare Services in Greece

The Greek NHS consists of 201 rural and 3 urban primary healthcare centres, 1478 positions in rural medicine and many outpatient departments in 140 public hospitals [11]. Primary health centres are composed of 1787 full-time salaried doctors (mainly general practitioners (GPs), specialists in internal medicine, paediatricians and dentists) and approximately 2414 other health professionals, most of them enjoying permanent tenure [11]. Thus, many small medical centres are often staffed by medical doctors without a specialty [11]. Significantly, only recently, young and inexperienced doctors have been gradually replaced by general practitioners, according to the national law 2519/97, which, however, was passed in 1997 [11].

From 1994 to 2009, several reform proposals were aimed at encouraging patients’ freedom of choice in health care, at introducing the family physician as the cornerstone of primary care system’s structure, and unifying primary care services [11], but eventually none were implemented [11].

Consequently, the approaching obstacles faced by patients in rural areas forcing them to seek quality healthcare services in urban areas, private practice, and EDs [11].

Furthermore, some of the main reasons for the high primary healthcare costs are, firstly, that most GPs, although they work for the NHS, are also private practitioners, and, secondly, the repetition of tests and prescriptions due to poor electronic medical record systems [11,12,13]. The latter is perhaps the most critical factor demonstrating the ineffectiveness of the existing control mechanisms of health insurance funds [11,12,13].

### 2.3. In-Hospital EMS in Greece

An ED may be considered the benchmark, by which the quality of a healthcare system is estimated [1]. EDs constitute the vital link between pre-hospital and in-hospital medical care that provides professional care at any time [14].

The most basics components of a well-functioning control ED are the presence of triage, supported by national clinical guidelines to help state EMS systems to standardise their patient care [1,5]. Overcrowding and misuse of EDs have established the need for development and introduction of triage protocols throughout the whole EMS system, to achieve appropriate emergency care for patients [14]. Although almost all European Members (24/27) record triage protocol compliance in their hospital, only 19 countries claim that triage protocols are used by physicians [14,15,16]. Furthermore, only 9/27 and 10/27 European Member States have approved guidelines standardised by national standards or electronic recording, respectively [14,15,16].

In Greece, the majority of in-hospital EMS settings use triage systems to prioritise incoming patients rapidly. The aforementioned systems are standardised at a national level. However, the networks of clinical information are underdeveloped [16] and triage protocols are often not used by physicians [14,15,16]. In most cases, patients presenting to an ED have typically first been assessed by a nurse [15,16]. Hence, the nurse is allowed to assign the patient to a queueing system [15,16]. The emergency cases in Greece are usually sorted into surgical or medical emergencies and appropriately treated by specialists. Most of the subspecialties are forced to make disposition decisions, based mainly on their expertise and intuition, unfortunately without the presence of a standardized set of uniform codes and guidelines [14,15]. In life-threatening situations, anesthetists are involved [14,15]. Usually, the anesthetists manage the in-hospital emergencies or act as part of the resuscitation team [14,15]. In 2003, a law was promulgated governing the EDs’ development and operation in public hospitals with more than 200 beds. In 2004, the Olympic Games emerged as an important tool of EDs’ renewal [8].

## 3. Does Economic Recession Lead to Healthcare Crisis? What the Numbers Tell Us

According to the latest census by the Hellenic Statistical Authority (2011), Greece has a population of 10,816,286 [17]. The Greek population is equivalent to 0.15% of the total world population [17]. 78.7% of the population is urban (8,764,013 people in 2018) and they often have difficulties in accessing healthcare services [17]. Greece is one of the countries hardest hit by the economic crisis that began in 2009, and which has forced millions into poverty [18,19]. According to estimates made by Eurostat in 2015, 35.7% of the population are at risk of poverty or social exclusion [18,19].

The economic recession had a particularly significant negative impact on the gross domestic product (GDP) in Greece, as well as on annual healthcare spending growth. The country’s GDP fell from 236 billion € in 2008 to 184 billion € in 2016, a value loss of 22% [18].

Before the crisis, total health expenditure was estimated at 8.6% of GDP in 2003. It rose further in 2009 reaching 9.9%, of which 5.3% and 4.5% was of public and private origin, respectively [19]. Notably, total health expenditure as a percentage of GDP was in line with the European average, but has decreased substantially since the crisis started [18]. The mean per capita expenditure on health services in Greece was about threefold lower than the mean amount for European Union (EU) (€26.2 vs. €75.8) in 2009 [18] and dropped further by 13% in the coming years (€23.1 in 2012). Additionally, the mean per capita healthcare expenditure suffered a decline from €2977 in 2009 to €1663 in 2015 with an overall decrease of 6.6% since 2009 [13,19,20].

For Greece, this drop signaled a fall in health spending [18]. According to Eurostat, the Greek healthcare system was allocated €8.5 billion in 2016, or 4.9% of GDP [21].

Total (outpatient) pharmaceutical expenditure has been estimated to have decreased by 32% (€2.1 billion), but this was to the benefit of the Social Health Insurance funds which are the main funders of these expenditures [19]. The largest reduction was in public pharmaceutical expenditure (and other non-medical durables), at 43.2%—from €5.2 billion (roughly 2.25% of GDP) in 2009 to €2.95 billion (or 1.53% of GDP) in 2012 [19]. According to the terms laid down by the Memorandum of Understanding (MoUs) that designate the country’s fiscal policies and which was signed in 2009 by the Greek governments, the International Monetary Fund (IMF), the European Central Bank (ECB), and the European Commission (EC), pharmaceutical expenditure should not have exceeded €2.44 billion in 2013 and €2 billion in 2014 [20]. If these thresholds were exceeded, clawbacks from the pharmaceutical companies would be used to balance the budget [20]. Moreover, out-of-pocket pharmaceutical expenditure increased as a percentage of total health expenditure—from 27.6% in 2009 to 28.8% in 2012 [19,22].

It is also accepted that the frequency with which patients access emergency care is usually more strongly associated with a country’s burden of diseases than its gross national income [5]. In fact, the economic recession has negatively affected the health status of the population in Greece [6,18]. The leading causes of death in the country are cardiovascular diseases, malignancies, and external causes of injury (accidents) and poisoning (suicides and homicides), which are responsible for 72% of all deaths across the world, in 2015 [23]. Moreover, premature mortality rates, mainly from cardiovascular diseases, are higher in Greece than the European average [23]. Likewise, age-standardised mortality from all causes is higher in Greece than in other European countries, which can be explained by the excess mortality from diseases of the circulatory and respiratory system [23]. Moreover, the numbers of infant and maternal deaths, and suicides and homicides (for males only) have also increased [23].

## 4. Impact of the Economic Recession on EMS Function

### 4.1. Impact of the Economic Recession on Pre-Hospital EMS System

Though Greece’s economic recession may be stealing the limelight, the National Health Service as well EMS has been majorly affected by austerity [24]. The major problems that emergency service providers face today are the following: unbalanced distribution of primary healthcare centres according to population densities, inadequate training of primary healthcare staff, inefficient communication and transfer protocols, understaffing of ambulance services and unpaid overtime, and unrepaired vehicles due to budget problems [24]. There are reports documenting that some of Athens’s ambulances have many hundreds of thousands kilometers on the clock, and many are temporally out of use as a result of a lack of spare parts. It has also been reported that, at night, only a few vehicles cover the needs of a high-density population of more than 4 million. The problem of inadequate ambulance services becomes more evident in some islands and remote rural areas (mountain villages). In these cases, private ambulances, EKAV helicopters, and taxis may be legitimate alternatives, depending on the severity and urgency of the disease [8]. Since the beginning of the economic recession, spending on health care has been deeply cut, taxes have been hiked, and pensions have been in a steady decline. The shortages of critical medical supplies reveal the true extent of the crisis [24,25]. In particular, healthcare expenditure fell by 25% since 2009, thus leading to significant healthcare equipment deficits [6,25]. A Greek study conducted at the beginning of economic recession revealed the scarcity of crucial items of equipment such as spirometers in rural primary care [12,24,25]. Specifically, only 4.6% of rural doctors had spirometers that were considered adequate for clinical use [12,24,25]. Furthermore, during the last decade, state-funded pensions have suffered significant reductions; wages have been lowered by 20% and higher co-payments—up to 25% of a drug’s purchase price—have led to patients struggling to pay for medications [6,11,13]. Crucially, treatment non-adherence has been associated with more exacerbations and hospitalisations annually. Hence, the burden of many chronic diseases seems to be affected by an economic recession [6].

Primary health care suffers from many weaknesses, including unmet health needs in parallel with unnecessary overuse of curative treatments and diagnostic services. Overall, according to Filippidis et al., the prevalence of unmet need for health care has significantly increased from 10.0% in 2010 to 21.9% in 2015 [22]. Moreover, some ethics-related issues come to the surface. Data support the contention that primary care doctors do not declare all private practice [11]. Interestingly, it has been documented that the private primary care sector in Greece absorbs more than 65% of total private health expenditure and private diagnostic centres generate substantial earnings [11].

According to a study by Karakolias et al., nowadays, most doctors consider that their salary is unfairly low and that were paid less than private sector counterparts [26]. Younger respondents highlighted the fact that current low salaries favour dual employment and claiming informal fees from patients [26]. Older respondents underlined the negative impact of low wages on productivity and quality of services [26]. Greek primary care doctors are dissatisfied with the current remuneration scheme [26]. Consequently, many doctors have left Greece since 2010, ending up in countries, where Europe’s economic turmoil has had less impact.

### 4.2. Impact of the Economic Recession on In-Hospital EMS System

#### 4.2.1. Overcrowding

EDs offer whole-day, free of charge, preventive, curative services responsive to emergencies as well as rehabilitation services—mainly to urban and semi-urban populations [27]. On the other hand, in primary care facilities, uninsured patients have to pay their medical bills for most care visits. The only exception to this rule is an expat employed in Greece, with a social security card (known as an AMKA), who pays for public health insurance. In 2016, the Greek government extended health coverage to uninsured people who are registered as unemployed as well as to refugees. Specifically, those who have less than 2400 euro per year of earned income are entitled to free health care, with the threshold rising according to the number of children in families.

In the years of crisis there has been a shift from the private to the public healthcare sector, as shown by an increase of 24% in the number of admissions to public hospitals from the very beginning of the crisis (from 2009 to 2010), that continued to rise in the first half of 2011 by 8% [11]. Conversely, it has also been documented that there was a decrease in admissions to private hospitals in the period 2009–2010 [10]. Out-of-pocket payments are high in care provided by the private sector. The reductions in health budgets—imposed after 2009—were accompanied by increases in the numbers of persons unable to access health care, particularly and vulnerable groups [28].

During the past few decades, a continuous rise in the rate of ED visits has been observed globally [14]. Emergency room (ER) crowding has become a widespread problem worldwide [29]. Crowding is defined as a situation in which the identified need for emergency services exceeds available resources for patient care in the ED, hospital, or both [29]. This increase is exacerbated by a hospital’s organisational problems, for instance, shortages of staff, laboratory, and admission delays. Accordingly, an ED becomes overcrowded, with inevitable consequences [14,27,30]. However, the extent of the workload currently has not been thoroughly evaluated in Greece, as patient data are not regularly recorded.

It has been reported that EDs accepted more than 5,000,000 patients per year [30]. This corresponds to the 40% of the total of patients who visit public hospitals. The body of aggregated literature strongly argues that ED overcrowding is associated with potentially poorer clinical outcomes, including mortality [29]. A primary factor that may cause crowding in Greek EDs is the inadequate staffing [23]. The Greek EMS system is characterised by a low density of nurses as well as high hospitalisation rates [23]. However, it is striking that Greece has one of the highest number of physicians (6.17 physicians per 1000 people, in 2013) in the world [23]. This number has increased after the crisis began, as in 2004 the figure was only 4.38 per 1000 people [11]. Although the density of physicians is one of the highest in Europe, high unemployment rates forced many doctors to seek work abroad. Without a doubt, the stress of this unsupportive work environment leads doctors to emotional burnout and depression [24].

At the begging of the economic recession, 48 hospital beds per 10,000 people were recorded [23]. This rate was higher than in other advanced countries such as the United Kingdom (39 beds), Italy (39 beds) and Spain (34 beds) [22,23]. Notably, to serve unexpected inpatient care, Greek public hospitals present a 14% excess bed capacity [31]. In 2011, the Ministry for Health and Social Solidarity announced its intention to reduce public hospitals and the proportion of beds available for general purposes in the country [8]. Hence, the number of public facilities and hospital beds has been declining over the last years, from 140 hospitals with 36,400 beds to 83 hospitals with 33,000 beds [8]. Taking into consideration that hospital bed shortages are factors that affect crowding, the future may be much worse than the past over time. In short, cutting the health workforce’s salaries, limiting recruitment of health personnel, and reducing procurement of medical supplies constitute a triple threat to the population’s health and well-being [24].

The ageing of the population as well as the upcoming economic and social implications of the aging process, raise concerns in all western countries, as they seem to have a considerable effect on healthcare systems leading to overcrowding. In Greece, the population aged over 65 will exceed 3.5 million in 2060, compared to 2.1 million in 2008 [23]. In other words, an impressive increase of 68% in the ageing population is expected, alongside with a 10% and 18% decrease in the young, aged 1–14, and productive people, aged 15–64, respectively [23].

#### 4.2.2. Effects of the Economic Recession on Cardiovascular Disease

With all Greece’s problems, raising taxes and cutting government spending has led to a sharp economic slowdown and higher unemployment. The unemployment rate in Greece the highest among the European countries, reaching 27% in 2014, and fell to 23.5% in 2016 [17].

Harm in coronary health cannot be ruled out during an economic recession and cardiovascular disease may be partly attributable to the consequences of unemployment. Remarkably, it has been reported that death rates by diseases of the circulatory system declined more slowly after the onset of the crisis than before [32]. Besides, coronary artery diseases are the most common cause of high death rates in emergency or home cases, since still two-thirds of all patients die before reaching the ED [33]. This impression is based on studies more than a decade old which found that among patient older than 55 years who died from cardiac arrest, 91% did so outside hospital [33]. In fact, when thrombolysis is required, survival is related to the “call to needle” time, which should be less than 60 min [33].

Likewise, the incidence of stroke in Greece is among the highest in the western world, with a very low proportion of surviving patients [34]. Stroke deaths in Greece reached 20,662 or 21.86% of total deaths [35]. These data rank Greece as 101th (highest mortality per capita) globally. Stroke mortality is as high as 130 per 100,000 in the general population [34,35]. According to data from the ViewCronos database, mortality for stroke in Greece is 50% higher than the mean mortality rates of the EU [34,35]. There are 3 different types of settings where a patient with stroke can be admitted to a Greek state hospital [34,35]. These include a medical ward (MW), a neurology ward (NW), and a specialised stroke bay (SB). The SB is a designated area for stroke care attached to an NW [34,35]. Nonetheless, currently, Greece has only 2 designated SBs, one in the capital of Athens and another in the co-capital, Thessaloniki [34,35]. The staffing ratio is approximately 8 to 10 patients to each nurse on all of the MWs and NWs, and the ratio is 6:1 on the SB unit [33,34]. The long-standing economic recession in Greece has resulted in “skeleton staff” throughout public hospitals where new job opportunities are scarce [34,35]. Additionally, there are no graduate stroke programs [34,35]. Greek nurses in general, and those working in stroke care individually are in need of greater educational support because stroke is not viewed as a high priority by healthcare policy makers [34,35]. Gioldasis et al., estimated that the in-hospital cost of stroke is characteristically dependant on the type of stroke [36]. The average cost of an ischaemic stroke calculated to be $3908 [36]. Patients with intracerebral hemorrhage incurred the highest costs of care, at roughly $5583 on average [35]. In contrast, patients with a lacunar stroke were the least costly, at approximately $2423 on average [36]. The cost of treatment has been reported to account for approximately 10% of bed-day expenses [36].

#### 4.2.3. Effects of the Economic Recession on the Prevalence of Accidents

Greece has the third highest rate of death due to car accidents. 77.4% of the 2500 fatal injuries due to car crashes happen far away from any healthcare institution, thus resulting in longer response times. Notably, the 66% of injured patients die during the first 24 h [37]. It has been recognized that Greece has the highest number of deaths from single vehicle road collisions in the EU [23]. This is a sobering thought, given that road accidents of this type accounted for 42% of road fatalities. However, some results also supported the idea that an economic recession led to a healthier lifestyle [6,32]. Laliotis et al., documented that deaths from vehicular accidents declined faster after the onset of the crisis, especially among men between the ages of 20 and 34 [32]. The continually increasing costs of fuel, taxation in combination with the rising costs of vehicle insurances, services, and toll fees forced many drivers to shift to cheaper means of transportation [38,39]. The recorded alcohol consumption per capita for the adult population in Greece decreased over the last three decades to a record low of 7.9 L per capita in 2010 [23].

Moreover, according to Pouliakas and Theodosiou, there seems to be a correlation between occupational accidents with factors such as low educational level, long hours at work, low family income, long-lasting unemployment, monotony, employees’ lack of satisfaction, and non-creative work in general [37]. In periods of prolonged economic downturn and weak financial performance, there is an increase in the frequency of occupational injuries [37]. The causes are found in the increased workload, pressing working conditions, employment insecurity, reduced investment for the decrease and elimination of occupational hazards, work-related stress, increased average age of employees, as well as the increased participation of migrants in the final product [37].

Furthermore, the incidence of fractures due to interpersonal violence increased during the period of the severe economic [39]. Moreover, a significant concern is the lack of appropriate equipment for surgical innervations, resulting in problematic curative healthcare services [29]. Consequently, the crisis directed surgical patients to the public healthcare sector only in cases of severe diseases [29].

## 5. The Refugee Crisis Challenges Greek EMS System

The unprecedented flow of migrants arriving in Europe over the last three years has caused major health consequences. Specifically, in June 2015, 124,000 migrants and refugees had arrived in Greece. This represents a 750% increase compared to 2014 [40]. The Greek Government on 1st March 2016 was forced to request emergency funds from the EU to provide shelter for the unexpected wave of refugees. The amount needed has been estimated at 480 million euros [39]. Additionally, it has been argued that more than 60,000 asylum seekers were trapped in Greece due to closed European borders [40]. Therefore, it is essential the country identifies practical long-term solutions to hosting approximately 40,000–60,000 refugees [41]. Overall, the dramatic increase in the number of asylum seekers and migrants entering the country resulted in major political, economic, social, and health dimensions [40,41]. Specifically, this influx created significant challenges for all national healthcare systems across Europe, and consequently in Greece [40,41].

In fact, hospitals are struggling to respond to the needs and demands of both local people and migrants, mainly due to a lack of resources as previously mentioned [40,41]. Whilst they theoretically have access to the treatment in public hospitals, in reality, access is difficult due to a general lack of medical and human resources [40,41]. Moreover, lack of precise mechanisms for coordination creates significant difficulties in the implementation of national health policy. Despite the fact that the Greek Social Security System is thought to cover unemployed or uninsured people it seems extremely difficult to cover any additional care expenses burden amid economic crisis [40,41]. This means that the vast majority of immigrants will seek medical help in the public sector only in emergency settings and for advanced illness [40,41]. To address these needs, healthcare providers should also be trained in applying integrated and culturally competent health care [40,41].

## 6. Conclusions and Future Directions

The Greek EMS system has been profoundly affected by the economic recession. As per capita health spending growth has slowed significantly since 2009, all major health spending categories have been affected to varying degrees. The primary healthcare network is characterised by inconsistency in the availability, accessibility, and quality of primary healthcare services between urban and rural areas. Reductions in health expenditure have led to inadequate health promotion services. Furthermore, the multi-factorial problem of in-hospital EMS overcrowding has rapidly deteriorated and resulted in severe safety issues for patients. There is also evidence that the economic crisis has substantially affected chronic patients’ access to healthcare services, jeopardising patients’ well-being. The current economic framework poses additional threats to the healthcare system and its sustainability. 

### Future Directions

Nevertheless, it is generally accepted that there are no “quick fixes”. Innovative operational and workforce models of care within EDs constitute a real solution. Urgent needs include the expansion of the primary medical network and outpatient departments, the development of the independent specialization or specialty of emergency medicine—highly trained physicians who will reduce the ED’s workload—and the adoption of a triage scale in all hospitals. The quality and efficiency of primary care could be empowered by introducing quality indicators, as well as the distribution of practice guidelines and clinical protocols for the most common diseases and health problems. Furthermore, development of an electronic file for recording patient data would automate EMS processes. Continuing medical education (CME) as a useful method of assessment is a valuable tool to improve guideline adherence and implementation in many diseases. Quality assessment and management systems should also be developed to monitor the changes. Effective enforcement of the aforementioned concerns is expected to have a favourable effect on the Greek primary healthcare system, and such proposals may be a first attempt to improve the current EMS system.
